# The Oscillopathic Nature of Language Deficits in Autism: From Genes to Language Evolution

**DOI:** 10.3389/fnhum.2016.00120

**Published:** 2016-03-18

**Authors:** Antonio Benítez-Burraco, Elliot Murphy

**Affiliations:** ^1^Department of Spanish Philology and its Didactis, University of HuelvaHuelva, Spain; ^2^Division of Psychology and Language Sciences, University College LondonLondon, UK

**Keywords:** autism, neural oscillations, RUNX2, evo-devo, biolinguistics, language evolution

## Abstract

Autism spectrum disorders (ASD) are pervasive neurodevelopmental disorders involving a number of deficits to linguistic cognition. The gap between genetics and the pathophysiology of ASD remains open, in particular regarding its distinctive linguistic profile. The goal of this article is to attempt to bridge this gap, focusing on how the autistic brain processes language, particularly through the perspective of brain rhythms. Due to the phenomenon of pleiotropy, which may take some decades to overcome, we believe that studies of brain rhythms, which are not faced with problems of this scale, may constitute a more tractable route to interpreting language deficits in ASD and eventually other neurocognitive disorders. Building on recent attempts to link neural oscillations to certain computational primitives of language, we show that interpreting language deficits in ASD as oscillopathic traits is a potentially fruitful way to construct successful endophenotypes of this condition. Additionally, we will show that candidate genes for ASD are overrepresented among the genes that played a role in the evolution of language. These genes include (and are related to) genes involved in brain rhythmicity. We hope that the type of steps taken here will additionally lead to a better understanding of the comorbidity, heterogeneity, and variability of ASD, and may help achieve a better treatment of the affected populations.

## Introduction

Autism spectrum disorders (ASD) are pervasive neurodevelopmental disorders involving several social and cognitive deficits. Usually, people with ASD exhibit stereotypical and repetitive behavior, an inability for social interaction, and communicative problems (Bailey et al., 1996). Interestingly, close connections have been made between ASD and specific language impairment (SLI; see Crespi and Badcock, 2008 for discussion and “From Language Deficits to the Brain in ASD” Section below). ASD involves atypical brain wiring during growth, which results in its distinctive cognitive profile, and which has been linked or associated to mutations in an extensive number of genes (Veenstra-VanderWeele and Cook, 2004). Recent advances in genome-wide technology have resulted in a long list of candidate genes for this condition (Geschwind and State, 2015). Although they point to specific pathways and neural mechanisms underlying its associated deficits (Willsey and State, 2015), the gap between the pathophysiology of ASD and genes still remains open, in particular regarding its distinctive linguistic profile (see Jeste and Geschwind, 2014 for discussion). In truth, the polygenism seen in ASD is somewhat commensurable with the polygenism displayed in language more generally. The goal of this article is to contribute to bridging this gap between genes and ASD, focusing on how the autistic brain processes language, particularly through the perspective of brain rhythms and how language evolved in the species.

Brain rhythms are primitive components of brain function. Because the hierarchy of brain oscillations has remained remarkably preserved during the course of mammalian evolution, it has been hypothesized that cognitive disorders can be conceived as variations (i.e., dysrhythmias or oscillopathies) within the network constellation that constitutes a universal brain syntax (see Cobb and Davies, 2005; Buzsáki et al., 2013 for discussion). And because brain rhythms are connected to some computational primitives of language (see Murphy, 2015a for discussion), we believe that interpreting language deficits in ASD as oscillopathic features is a potentially fruitful way to construct successful endophenotypes of this condition. Current understanding suggests that ASD is characterized, amongst other things, by asynchronous neural oscillations (Tierney et al., 2012), although as discussed below a number of studies have reported both underconnectivity and overconnectivity in the autistic brain. We hope that the type of steps taken here will additionally lead to better understanding of the comorbidity, heterogeneity, and variability of ASD, and may help achieve a better treatment of the affected populations. Since children can progress from one sub-type of disorder to another during development, long-term studies of children with ASD involving perennial oscillopathic monitoring could ideally provide a more accurate diagnosis (refining the disorder boundaries) and prognosis. Additionally, a comprehensive picture of the rhythms and networks implicated in linguistic deficits in ASD should also contribute to the growing understanding of the human cognitive phenotype, set against an evolving dynamic model of mental computation. This is why we also expect our approach to cast light on the neurobiological basis of language and the way in which language evolved in our species. In doing so, we will heavily rely on our attempts of translating language into a grammar of brain rhythms, as developed in Murphy (2015a), which represents a new approach to the problem that the categories of linguistic theory do not easily map onto clinical typologies. This also builds on ongoing developments in neurolinguistics which move beyond the outdated classical production-comprehension division, and which bring to the forefront of discussion the dynamics of brain networks; that is, the collections of brain areas jointly engaged by some cognitive operation (Fedorenko and Thompson-Schill, 2014). But we will also rely on an evolutionary-developmental (evo-devo) perspective in our ongoing research into the origin and development of language, which aims to find a robust link between the changes that occurred in the human brain during our recent speciation and the changes occurring in the child brain during language growth. According to our view, our language-readiness (i.e., our species-specific ability to acquire and use languages) depends greatly on a proper pattern of cortical inhibition and a specific pattern of long-distance connections across the brain (which was brought about by mutations in genes controlling the development of the skull and the brain), that enable us to form and exploit cross-modular concepts (see Boeckx and Benítez-Burraco, 2014a,b; Benítez-Burraco and Boeckx, 2015a for details), both of which are aspects that are targeted in ASD (Khan et al., 2013; Zikopoulos and Barbas, 2013).

The article is structured as follows. First, we provide a general account of language deficits in ASD. Afterwards we describe the anomalies in brain activity observed in ASD and advance a tentative oscillopathic model of language deficits in ASD. Then we move to the genes, focusing on candidates for ASD that may help to explain this abnormal profile of brain rhythmicity. The last section of the article examines the whole case for the oscillopathic nature of language deficits in ASD from an evolutionary perspective, arguing that the overrepresentation of candidates for ASD among the genes that changed after our split from extinct hominins may help understand how our language-readiness evolved, but also the nature and the prevalence of ASD among modern populations.

## From Language Deficits to the Brain in ASD

### The Language Phenotype in ASD

Language deficits in ASD are not always confined to the pragmatic component of language, which is also compromised in other intellectually impaired children (Abbeduto and Hesketh, 1997). On the contrary, some children with ASD undergo a real linguistic regression from age 12–24 months (Lainhart et al., 2002; Lord et al., 2004), or never acquire functional speech (Tager-Flusberg et al., 2005). Moreover, phonological and morphosyntactic problems are observed in nearly one third of children with ASD (Tager-Flusberg and Cooper, 1999; Rapin and Dunn, 2003; Tager-Flusberg and Joseph, 2003). Interestingly, as noted, ASD is sometimes comorbid with language disorders like SLI (Norbury, 2005; Tager-Flusberg, 2006). It has been hypothesized that a core language deficit exists in ASD, although this is difficult to prove because of the noteworthy variability in the linguistic and communicative abilities of people with ASD, and of the masking effect of their variable IQ and the degree of functionality achieved in the domain of language (see Volden and Lord, 1991, on MLU, and Roberts et al., 2004 on person and tense marking). The impairment of the oromotor function has been claimed to account for expressive language deficits in a subgroup of people with ASD (Belmonte et al., 2013). Nonetheless ASD also entails problems in language comprehension. Moreover, Theory of Mind and related pragmatic faculties do not amount to an explanatory account of language deficits in ASD, and even documented cases of ASD which do not result in formal language impairments still display patterns of linguistic processing and acquisition deviating from typically developing (TD) controls (Howlin, 2003; Bourguignon et al., 2012).

Among the distinctive profile of atypical language development in children with ASD, one observes that syntactic complexity grows at a similar rate than in children with Down syndrome (although the former employ fewer functional words; Tager-Flusberg et al., 1990). Additionally, children with ASD rely less than their unaffected peers on prosodic cues to disambiguate sentences (Diehl et al., 2015), and less on semantic plausibility to understand passives (Paul et al., 1988). It has been further claimed that they integrate semantic information differently when interpreting syntactic constructions (Eigsti et al., 2007) and that semantic knowledge seems to consolidate in a dissimilar manner. Accordingly, they choose less prototypical words and are less primed by semantically-related words; moreover, even if they seem to be constrained by the “Principle of Mutual exclusivity” (i.e., “expect that each thing in the real world is referred to with one and the same word only”), they are less shape-biased than controls when categorizing items (Dunn et al., 1996; Kamio et al., 2007; Tek et al., 2008). Ultimately, children with ASD exhibit linguistic features that are not observed (or that are much less frequent) in children without ASD, such as echolalia (Tager-Flusberg and Calkins, 1990) or neologism formation (Volden and Lord, 1991; Eigsti et al., 2007). Overall, language acquisition in ASD proceeds in a more scattered way (e.g., the structure of new sentences is less predictable based on previous constructions; Eigsti et al., 2007), whereas the degree of variability and heterogeneity of language growth is greater than in the TD population (see Kjelgaard and Tager-Flusberg, 2001; Luyster et al., 2007; Norbury et al., 2010 on vocabulary development). As pointed out by Eigsti et al. (2007: p. 688), in ASD we often observe a “dramatic variability across and between social, cognitive, and language domains, [while] in studies of typical development […] there is generally a much smaller range of individual differences”.

Importantly, ASD also entails differences regarding the way in which other cognitive abilities are put into use during language acquisition and usage. For instance, because of the reduced role played by semantic priming during word learning, some children with high-functioning ASD rely more than controls on their (enhanced) capacities for auditory/phonological processing and for statistical/associative learning (Tager-Flusberg, 2006; Kuhl, 2007; Preissler, 2008). At the same time, children with ASD showing deeper language deficits are thought to suffer from some deficit in phonological processing (Lindgren et al., 2009; see Norbury et al., 2010 for a discussion). Delays, asynchronies, and/or deviances are also observed at this cognitive level throughout development. For instance, because word learning depends more on an (enhanced) capacity for associative learning (at least in some children with ASD), it has been claimed that in ASD the declarative memory may play a more central role in language acquisition (Walenski et al., 2006). Similarly, problems with syntax are suggestive of an impairment of the procedural memory. Language impairment in ASD also involves problems with binding, relative clauses, *wh*-questions, raising and passives (Perovic and Janke, 2013).

In DSM-V, unlike DSM-IV, the number of ASD symptom domains has been reduced to two: “Social communication domain” (created by the merger of key symptoms from the DSM-IV Social and Communication domains) and “Fixated interests and repetitive behavior or activity”. Although language deficits are no longer explicitly defined as a central feature of ASD (because deficits in communication are intimately related to social deficits), an examination of the fixated interests and repetitive behavior described in DSM-V reveals that “stereotyped or repetitive speech” is nevertheless attributed a major role in ASD criteria; pronoun reversal, abnormal self-reference, repetitive vocalizations, unusually formal language, echolalia and neologisms are considered criteria exemplars. Moreover, we believe that this does not preclude the centrality of language deficits within the ASD phenotype. In truth, in the TD population communication is always intimately related to the social domain. But at the same time, it makes sense to claim that human beings are endowed with a special faculty for acquiring and using languages even if it relies on cognitive devices that are not specific to language (Hauser et al., 2002; Boeckx, 2011). Likewise, we believe that language deficits in pathological conditions can be described and interpreted on their own, in spite of the fact that they result from the impairment of biological components that are not specific to language (see Benítez-Burraco, 2016 for discussion).

### The Linguistic Brain in ASD

Recent neurobiological findings give support to these perspectives. Accordingly, many intriguing structural differences are observed in the brains of people with ASD when they are compared to non-affected subjects. These include regional differences in brain volumes, variations in gray matter and white matter volumes and thickness across many brain regions, and differences in inter- and intra-hemispheric connection patterns (reviewed in Stefanatos and Baron, 2011; Bourguignon et al., 2012). As expected, most of these regions and nerve tracks are relevant for language processing. For example, children with ASD show an increment of gray matter density in the primary and associative auditory and visual cortices (Hyde et al., 2010). Likewise, high-functioning children with ASD have smaller gray matter volumes in the frontostriatal regions, whereas children with Asperger syndrome (which exhibit milder language deficits) still show reduced volumes of the caudate and the thalamus (McAlonan et al., 2008). As Radalescu et al. (2013) discuss, fronstostriatal connectivity is a core component of healthy linguistic cognition, and, as explored below, the thalamus may be also. Finally, microstructural anomalies and reduced lateralization patterns are characteristically observed in the arcuate fasciculus of people with ASD (Fletcher et al., 2010), suggesting that language impairment in these individuals may result in part from a constraint of the integrative processes during development (Schipul et al., 2011). Not surprisingly then, functional differences important for language processing have been attested as well in the brain of people with ASD. Hence, in children with ASD networks involved in the statistical analysis of speech respond abnormally to artificial languages (Scott-Van Zeeland et al., 2010b): this finding can be related to their attested deficits for implicit learning (Mostofsky et al., 2000). In such cases, the basal ganglia and the left temporo-parietal cortex are impaired (Scott-Van Zeeland et al., 2010a), which reinforces the view that a cognitive hallmark (or endophenotype) of ASD is a dysfunction of procedural memory. Likewise, Courchesne and Pierce (2005) describe ASD neurocognition as the frontal cortex unconsciously “talking to itself” due to the impairment of normal language functions. In ASD, the frontal cortex networks are usually underactive during language comprehension tasks whenever sentences are not congruent with reality, suggesting that the integration of linguistic and encyclopedic knowledge is also impaired in this condition (Tesink et al., 2011).

Finally, regarding the atypical course of language development in ASD, Stefanatos and Baron (2011: pp. 262–263) point out that “functional anomalies early in development can have crucial implications for neural networking and environmental transactions that, in turn, prompt other potentially more widespread perturbations in cognitive structure or neural architecture”. As noted by Crespi and Badcock (2008: p. 244), deficits in the so-called maternal brain, largely the neocortex, alongside normal functioning in the paternal brain, largely the limbic system, can “lead to the loss of language, mental retardation, and repetitive behavior typical of infantile (Kanner) autism, whereas increased paternal-brain effects, but relatively spared maternal-brain function, may lead to high-functioning autism or Asperger syndrome”. Not surprisingly, some candidate genes for ASD are subject to imprinting (see for example Bonora et al., 2002).

That said, ASD cannot simply be reduced to deficient neural development and connectivity; Hahamy et al.’s (2015: p. 302) study of resting-state activity in adult ASD subjects revealed both increased and decreased intra- and inter-hemispheric connectivity. To account for this, they suggest that ASD can be characterized by idiosyncratic distortions of functional connectivity patterns, since the “magnitude of an individual’s pattern distortion in homotopic interhemispheric connectivity correlated significantly with behavioral symptoms of ASD”. In the next section, we will focus on brain rhythmicity and will try to (re)interpret the linguistic deficits observed in ASD in terms of abnormal patterns in the integration of brain oscillations. As expected, ASD also involves differences at the functional level during language processing by the brain, for example, anomalies in activation patterns or changes in mismatch negativity responses to linguistic elements (reviewed in Stefanatos and Baron, 2011: pp. 259–262), and an abnormal pattern of brain rhythmicity.

## From Brain Rhythmicity to Language Deficits in ASD

As noted above, ASD has been associated with a number of abnormal structural and functional patterns, which likely contribute to the emergence of the disorder (see also Welsh et al., 2005; Pineda et al., 2012). But if Hahamy et al. (2015) are correct in pointing to the idiosyncratic nature of connectivity patterns in ASD, functional localization studies may not provide the right basis from which to construct linking hypotheses between neural and linguistic operations. According to Uhlhaas et al. (2010), we should expect a strong relationship between the emergence of altered oscillatory patterns during childhood and the appearance of a number of neurocognitive disorders at distinct developmental stages, to the extent that brain oscillation self-regulation has been proposed as a potential treatment for ASD (see Pineda et al., 2012 for details). A brain that grows differently and assumes an unusual size is also differently wired and, ultimately, exhibits altered oscillatory behavior, which we will argue alters their cognitive phenotype (see Buzsáki et al., 2013 for how rhythms can be both preserved and altered with changing brain size). Accordingly, we believe that ASD should not only be seen as a cognitive, rather than purely social disorder (as argued by Bourguignon et al., 2012), but it should also be seen more specifically as an oscillopathic one. Under our view, subjects with ASD might therefore “*construe* language differently, reflecting a linguistic style different from that inherent in neurotypical cognition” (Hinzen et al., 2015). Once ASD is seen as an oscillopathy, and once brain dynamics can be shown to be a plausible candidate for linguistic computation (Murphy, 2015b), various predictions can be generated about the etiology of language-related disorders, and specifically, our understanding of language deficits in ASD (as reviewed above) and why language in ASD subjects is indeed construed differently (as suggested by the impairments in communication and pragmatic cognition documented in Mandy and Skuse, 2008) will surely develop.

### Language Processing under an Oscillatory Lens

**Figure 1:**
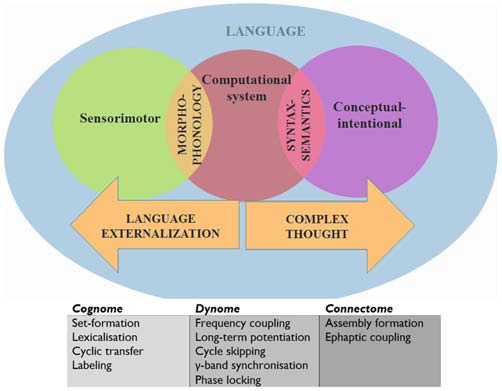
**A schematic view of language representing the systems and interfaces of interest**.

Before generating such predictions we will provide with a brief outline of our translational proposal of language into a syntax of brain oscillations. In doing so we will rely on the model of the human cognome-dynome outlined in Murphy (2015a), where “cognome” refers to the operations available to the human nervous system (Poeppel, 2012) and “dynome” refers to brain dynamics (Kopell et al., 2014). In this model various cross-frequency couplings and regions were attributed distinct computational roles under a research program termed “Dynamic Cognomics” (Figure 1). As Petersen and Sporns (2015: p. 207) emphasize, most accounts of cognition have “focused on computational accounts of cognition while making little contact with the study of anatomical structures and physiological processes”. Since “[t]he fact that a theory is computationally explicit does not automatically render it biologically plausible” (Bornkessel-Schlesewsky et al., 2014: p. 365), Murphy (2015a) attempted to correct for this imbalance by decomposing the computational operations of language down to a small set of (potentially generic) sub-operations, attempting to achieve an appropriate level of granularity from which computational-implementational connections could be constructed. Considerations of systems level computation were addressed, departing from the standard focus on single-neuron computation (Fitch, 2014). By connecting these dynomic insights to the “connectome” level, a more powerful computational model of dynamic brain activity was proposed. This model embraced the minimalist conception of language (Chomsky, 1995; Narita, 2014) as a *computational* system linking generated, hierarchically structured expressions to two interfaces; the *conceptual-intentional* system (responsible for “thought” and interpretation) and the *sensorimotor* system (responsible for externalization; Figure 1).

The computational system requires some elaboration. This is composed of the operations Merge (which generates sets and moves objects to different positions), Label (which assigns a built set an independent categorial identity, such as a Noun Phrase or Tense Phrase), Agree/Search (which establishes relations between objects, such as in Person or Gender agreement; in the sentence “There *seems* to be likely to be *a man* in the garden”, the italicized constituents exhibit syntactic covariance, with their number features agreeing) and Spell-Out/Transfer (the part of the memory buffer which sends structures to be externalized and interpreted in “chunks”, or “phases”; see Narita, 2014).

**Figure 2:**
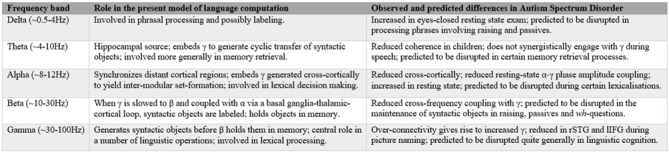
**Summary of the present cognome-dynome model of linguistic computation and the observed differences in autism spectrum disorders (ASD); rSTG denotes right superior temporal gyrus, lIFG denotes left inferior frontal gyrus (see Cornew et al., 2012; Buard et al., 2013; Berman et al., 2015; Jochaut et al., 2015; Kikuchi et al., 2015; Murphy, 2015a)**.

We will assume, following Murphy (2015a) and Theofanopoulou and Boeckx (forthcoming), that the *α* band embeds *γ* rhythms generated cross-cortically, the dynomic realization of inter-modular conceptual combinations, or set-formation (“Simplest Merge”, for Epstein et al., 2015). This form of variable binding may also arise from precisely controlled recurrent interactions between the basal ganglia and prefrontal cortex (Kriete et al., 2013). This perspective is supported by recent findings that *α* is responsible for visuo-spatial feature-binding, a form of representation “merging” (Roux and Uhlhaas, 2014). Since syntactic theory typically assumes that set-formation purely involves the combination of two representations without modification of either (Narita, 2014), these oscillatory mechanisms are perhaps the best implementational candidates. We will assume also that Spell-Out/Transfer is realized through embedding such *γ* rhythms inside the *θ* band, the source of which is found in the hippocampus. This perspective is supported by the recent finding that *γ* bursts “reflect the binding of temporal variables to the values allowed by constraints introduced by temporal expressions in discourse” (Brederoo et al., 2015) and by Meyer et al.’s (2015) EEG study suggesting that frontal-posterior *θ* oscillations reflect memory retrieval during sentence comprehension. Murphy (2015a) speculated that Transfer is also likely supported by the corpus callosum, following insights in Theofanopoulou (2015). More broadly, *γ* has been associated with lexical processing (Hannemann et al., 2007). Finally, we will assume that labeling (holding in memory one of the items before coupling it with another to generate an independent syntactic identity) involves the slowing down of *γ* to *β* before *β*-*α* coupling, implicating a basal ganglia-thalamic-cortical loop (see Hyafil, 2015 for discussion of the different types of cross-frequency coupling, such as phase-amplitude, phase-phase, and phase-frequency coupling). Assume also that Agree/Search is implemented via cross-cortical evoked *γ* due to the role of this band in attention and perceptual “feature binding” (Bartos et al., 2007; Sohal et al., 2009) and the distributed nature of the inter-modular representations Agree/Search operates over (see Figure 2 for predictions about rhythmic disruptions for agreement relations). Overall, we find this model compatible with recent findings that damage to the basal ganglia and thalamus can lead to various forms of aphasia and other linguistic deficits (Alamri et al., 2015); in turn contributing to an emerging rejection of the classical Wernicke-Lichtheim-Geschwind approach to neurolinguistics (Hagoort, 2014). This is why we expect it to contribute as well to a better understanding of language deficits in ASD.

As we have shown, decomposing linguistic processes into generic sub-operations permits a certain degree of alignment between the basic computational properties of the human nervous system and the neural oscillations which are increasingly being theorized as having major functional roles in memory, attention and perception. The next task is to use these cognome-dynome linking hypotheses to interpret the range of complex data accumulated from electrophysiological and magnetoencephalographic studies of ASD.

### Brain Rhythmicity and Language Processing in ASD

Dynomic investigations of ASD are still relatively young, but we believe enough has been learned to begin the construction of an oscillopathic model of this condition. The first physiological study of brain connectivity in ASD children under conscious conditions was conducted by Kikuchi et al. (2013), revealing aberrant brain activity and a rightward-lateralized neurophysiological network in ASD not present in TD children. They detected increased *γ* power—claimed to be related to the degree of developmental delay (Orekhova et al., 2007)—but reduced *α* and *β* (see also Cornew et al., 2012 for increased *δ*, *α* and *θ* in resting state exams). Similar *γ*-related findings were reported by Rojas et al. (2008), who argued that “gamma-band phase consistency … may be [a] potentially useful [endophenotype] for autism”. The central role attributed here to *β* in storing and sustaining multiple items in memory (part of labeling) generated by fast *γ* supports Bangel et al.’s (2014: p. 202) interpretation of their MEG study of number estimation in ASD. They found reduced long-range *β* phase synchronization in ASD subjects at 70–145 ms during the presentation of globally coherent dot patterns, providing “the first evidence for inter-regional phase synchronization during numerosity estimation, as well as its alteration in ASD”. If these patterns reflect problems with inter-regional communication, this would lend weight to the present view that *β* is deployed in the extraction and labeling of meaningful units (in the case of Bangel et al., 2014 the units included animal pictures), centered on basal ganglia activity in particular, which is strongly implicated in *β* activity by Khanna and Carmena (2015). Since children with ASD often struggle with communicative intent, the finding that imperative pointing implicates greater cross-cortical *β* activity than declarative pointing (Brunetti et al., 2014) would seem to support the current dynomic model, with Hinzen and Sheehan (2013) proposing a strong connection between linguistic cognition and imperative gestures. More generally, the disruption of rhythmic coordination (the basis of neural network communication, mediated through the synchronization of presynaptic potentials in a given neuronal population, enhancing postsynaptic impact on the target region; Donner and Siegel, 2011) seen in ASD supports the image of this condition as an oscillopathic disorder. Relatedly, as noted above, ASD has “naturally suppressed private speech”, which Frawley (2008: p. 269) claims are “best understood in the context of the control processes of cognitive-computational architectures”. We believe such control processes should in turn be best understood in terms of cross-frequency couplings between rhythms of distinct cortical and subcortical regions; a form of dataflow management (Pylyshyn, 1985); see Figure 2 for a summary.

Chattopadhyaya and Cristo’s (2012) exploration of GABAergic circuit dysfunctions in ASD additionally reinforces the central connectomic role attributed to these interneurons in Murphy (2015a). Numerous studies have discovered GABA_A_ and GABA_B_ receptor alterations in the brains of people with ASD (Fatemi et al., 2010; Oblak et al., 2010), with emerging developments in electron microscopy, permitting the 3D modeling of dendritic networks, likely being able to contribute to a development of these connectomic topics (Fua and Knott, 2015). Additionally, the noted altered *γ* activity detected in ASD children by Kikuchi et al. (2013) may have arisen from GABAergic or glutamatergic mediator system disturbances, claimed to be involved in producing this rhythm (Sohal et al., 2009). With Blatt et al. (2001) reporting significantly reduced GABA_A_-receptor binding in high hippocampal binding areas in ASD subjects, this may suggest deviant applications of syntactic Transfer operations in this condition. This perspective is supported by the following data: the smaller corpus callosum typically found in autistic brains (Waiter et al., 2005; Alexander et al., 2007); the reduced connectivity between left-anterior and right-posterior areas found in Kikuchi et al.’s (2015) MEG study of children with ASD, who exhibited a decrease in *θ* coherence; and the reduced *θ* inter-regional synchronization during a set-shifting task in children with ASD (Doesburg et al., 2013; although much more needs to be learnt about the rhythmogenesis of Transfer operations). Further, because the narrow syntactic operations of set-formation, Label and Transfer appear to be preserved in language disorders, we would predict that any dynomic variations detected in ASD would most likely reflect representational/conceptual and interface disruptions. For instance, although the dynomic basis has not been investigated, it is well documented that individuals with ASD have difficulty understanding abstract concepts, metaphors, and often cannot plan ahead and consider multiple options before selecting an appropriate response (Dodd, 2005: p. 47; Jordan, 2010).

Murphy (2015a) also discussed the over-arching role of the “Communication through Coherence” (CTC) hypothesis in rhythm synchronization (Fries, 2005). Coherent brain oscillations are a central mechanism for carving the temporal coordination of neural activity in a global network. More recent work by Fries (2015) expands on the general claim that synchronization affects communication between neuronal groups. Communication, or “the transfer of one representation in a presynaptic, or sending, group to a new representation in a postsynaptic, or receiving, group” (Fries, 2015: p. 220), is the process that implements neural computations and thus creates novel representations. If communication is disrupted, representation construction is consequently disrupted too, as in cases of rhythmic disruption in, for instance, monkey visual cortex (Tan et al., 2014). The emerging literature reviewed here suggests that this is precisely what happens in autistic brains. Neuronal communication, contrary to classical conceptions, is not limited to structural anatomical determination, but can also be achieved through emergent dynamic activity of neuronal groups (that is, at the level of the dynome). Rhythmic synchronization is widespread across the nervous system, and “[p]atterns of synchronization change dynamically with stimulation and behavioral context in a way that strongly suggests that selective coherence implements selective communication” (Fries, 2015: p. 222). As Uhlhaas et al. (2010) also argue, CTC suggests that neural synchrony is not an epiphenomenon but rather an integral part of cortical network functioning.

To take a case in which this rhythmic coherence is disrupted, we can turn to the abnormal forms of semantic organization documented in ASD (Harris et al., 2006). An MEG study by Braeutigam et al. (2008) recorded responses in adults with ASD to sentences ending with a semantically incongruous word. N400 responses following the incongruous word were weaker over left temporal cortices, and late positivity component responses to incongruous words and long-latency *γ* oscillations following congruous words were stronger in subjects with ASD relative to TD controls over prefrontal and central regions. The long-lasting *γ* possibly indicates “unusual strategies for resolving semantic ambiguity in autism” (Braeutigam et al., 2008: p. 1026), and this finding may relate to the claim that an inability to delimit activation within an abnormally wired network is a core neural marker of ASD (Polleux and Lauder, 2004). There may consequently be a lack of spatio-temporal and rhythmic activation constraints in ASD (possibly caused by an enlarged braincase, discussed below); a suggestion which speaks to particular communication deficits noted in ASD, such as general difficulties with spoken language and gestures, problems initiating and sustaining suitable conversation, and the use of inappropriate, repetitive speech (Lord et al., 2000). Gandal et al. (2010) discovered reduced *γ* phase-locking across hemispheres in ASD subjects during auditory pure-tone presentation, while induced and evoked *γ* were not significantly different from TD adults, again suggesting that problems with frequency synchronization is a central neural characteristic of ASD (see also Wilson et al., 2007 and their MEG study reporting reduced left-hemispheric steady state *γ* in adolescents with ASD in response to non-speech sounds). A more recent pure-tone study by Edgar et al. (2015) found pre-stimulus abnormalities across multiple frequencies for auditory superior temporal gyrus processes in ASD, followed by early high-frequency, and then low-frequency, abnormalities. For our purposes, the conclusion of Edgar et al. (2015: p. 395), that “elevations in oscillatory activity [suggests] an inability to maintain an appropriate “neural tone” and an inability to rapidly return to a resting state prior to the next stimulus”, speaks to the present hypothesis that ASD may be characterized by abnormal applications of language-related processes of representation maintenance (including labeling).

Deficits in the rhythmic profile of the core syntax-semantics regions were also found in a picture-naming task conducted by Buard et al. (2013), which used MEG and observed reduced evoked high *γ* in the right superior temporal gyrus and reduced evoked high *β* and low *γ* in the left inferior frontal gyrus in subjects with ASD compared to TD controls. Resting-state MEG data collected from adolescents with ASD also demonstrated that alterations of functional connectivity are dependent on region and frequency, and that frontal over-connectivity is “expressed in the gamma band, whereas posterior brain regions exhibit a disconnection to widespread brain areas in slower delta, theta and alpha bands” (Ye et al., 2014: p. 6062). In addition, resting-state *α-γ* phase amplitude coupling, a basic process in TD children, was recently found to be abnormal in children with ASD (Berman et al., 2015). Jochaut et al. (2015) also discovered atypical coordination of cortical oscillations in autistic linguistic cognition, combining EEG and fMRI to show that *γ* and *θ* cortical activity do not synergistically engage in response to speech. Oscillation-based connectivity between auditory and other language cortices was also found to be altered in ASD, compromising the mapping between sensation and higher-level cognitive representations.

We believe that these speech deficits in ASD can be investigated in oscillopathic terms. It has recently been shown, for instance, that there exists a strong correspondence between the average length of speech units and the hierarchy of cortical oscillation frequencies. Syllable sequences and phrases correspond with *δ*, syllables correspond with *θ*, and phonetic features correspond with *γ* and *β* (Schroeder et al., 2008; Giraud and Poeppel, 2012). These rhythms reflect a computational mechanism such that the brain “sets time intervals for analysis of individual speech components by intrinsic oscillations pretuned to an expected speech rate and retuned during continuous speech processing by locking to the temporal envelope” (Poeppel and Hickok, 2015: p. 252). In general, low frequency oscillations such as *δ* and *θ* parse speech streams into temporal units of granularities determined by the particular rhythm, while high frequency oscillations appear to decode these streams and access stored templates from memory. As a result, and considering the above findings of degraded *θ* and *γ* synergy in ASD, it may be the case that the lack of coherence between low and high rhythms leads to the documented problems with speech perception, tone recognition, and parsing phonemic representations at the rate seen in TD controls.

### Towards an Oscillopathic Characterization of Language Deficits in ASD

It seems, then, that a combination of slow, “global” oscillations and faster, “regional” oscillations can be impaired in ASD (Kikuchi et al., 2015), and that the coupling of such rhythms can also be impaired as a result. Even when hyperconnectivity is detected, as in Ye et al. (2014), dramatic losses of synchrony in the face of such increased oscillatory activity (documented, for instance, by Castelhano et al. (2015) in their EEG study of disrupted perceptual coherence) presents strong evidence that synchrony underlies central coherence. Since these cases of brain rhythm abnormalities likely result from the neural mechanisms underlying ASD, the spectral and spatio-temporal content distinguishing such rhythms can provide pertinent data for understanding its neurophysiological basis and oscillopathic profile. The idiosyncratic nature of connectivity patterns documented in ASD leads us to suggest that localization studies need to be supplemented by a (computationally explicit and informed) oscillopathic perspective.

The present perspective is therefore something of a refinement and expansion of Brock et al.’s (2002) seminal temporal binding hypothesis. In response to the wealth of data suggesting that ASD subjects showed “weak central coherence” (a bias toward piecemeal, and not configurational, processing), Brock et al. (2002) suggested that this feature arises from temporal binding deficits and a reduction in *γ* synchronization between local networks processing discrete visuoperceptual features. The picture, as we hope to have shown, has become more complicated in the intervening years, but our oscillopathic model should nevertheless be placed within the same tradition as Brock et al. (2002).

Finally, given that all of the oscillatory mechanisms we have invoked to construct our initial model of linguistic computation in Murphy (2015a) are general to a number of distinct cognitive systems, the oscillopathic profile we have constructed here is likely not unique to the language deficits in ASD. We would further predict that slight modifications to the rhythmic and connectivity patterns discussed above would result in different symptoms; for instance, when set at a particular level of disruption the oscillations responsible for various attentional mechanisms might lead to language-related attentional problems, but when disrupted in a different manner difficulties attending to socially relevant information might arise instead. Although we have restricted our attention to linguistic deficits, we see no reason to assume that our oscillopathic approach cannot also be fruitfully applied to an understanding of pragmatic deficits in ASD.

Overall, it seems that language deficits in ASD (as ASD itself) can be properly characterized as an oscillopathic condition. We believe that this translational effort, which aims to link the ASD dynome and cognome following the lines of Murphy (2015a), may result in a better understanding of language processing by people with ASD. Now we turn to genetics, which provides additional support for this view.

## ASD-Related Genes and Some Evolutionary Concerns

As noted in the introduction, the number of candidate genes for ASD has been growing over the time (Geschwind and State, 2015); at the same time, genetic studies also suggest that many of these candidates are related to specific pathways and aspects of brain function associated with susceptibility to ASD. Also several candidates for language impairment in ASD have been identified to date. Among the most promising genes one finds *MET* (Campbell et al., 2006), *CTTNBP2* (Cheung et al., 2001), *EN2* (Benayed et al., 2005), *NBEA* (Castermans et al., 2003), *HRAS* (Comings et al., 1996) and *PTEN* (Naqvi et al., 2000). It is possible too that somatic mutations affecting a subset of neurons cause ASD and language deficits in ASD (Poduri et al., 2013; Sahin and Sur, 2015). In the first part of this section we will focus on genes related to networks and pathways that seem to be crucial for the maintenance of the adequate balance between neuronal excitation and inhibition and more generally, of brain rhythmicity. In the second part we will re-examine candidate genes for ASD from an evolutionary perspective in the context of language evolution studies with a special focus on brain connectivity and function.

### ASD-Candidates and Brain Rhythmicity

As noted above, brain oscillation components and patterns are highly heritable traits, to the extent that we should expect that differences in cognition and behavior result in part from genetic variation affecting oscillatory activity. However, we don’t know how pathogenic genetic diversity results in altered pathological patterns of brain oscillations and in desynchronization of neuronal activity. Here we will provide with some recent insights regarding ASD (and language). Because of the concerns discussed in the previous section, candidate genes for ASD that are related to GABAergic activity are of great interest for us. For instance, the loss of *Mecp2* from GABAergic interneurons results in ASD-like repetitive movements and auditory event-related potential deficits in mice (Goffin et al., 2014). Importantly, in response to auditory stimulation *Mecp2*^+/−^ mice recapitulate specific latency differences as well as select *γ* and *β* band abnormalities associated with ASD that may help to explain high-order deficits in this condition (Liao et al., 2012). ASD has been associated as well to mutations in *GABRB3* (this gene encodes the β-3 subunit of the GABA receptor A; Cook et al., 1998; Shao et al., 2002, 2003). Interestingly, differences in the expression levels of genes encoding some of the GABA_A_-receptor subunits (particularly of β2 and β3) has been related to differences in the rhythm of hippocampal pyramidal neuron firing and the activity of fast networks (Heistek et al., 2010). More generally, genetic variation in GABA_A_ receptor properties have been linked to differences in *β* and *γ* oscillations, plausibly impacting on network dynamics and cognition (Porjesz et al., 2002). Documented alteration of the GABA catabolism also results in brain and behavioral anomalies that mimic the problems observed in people with ASD, including language deficits (Gibson et al., 1997; Pearl et al., 2003). Moreover, through exploring the functional relationships between ASD candidate genes by using the *BrainSpan* human transcriptome database, Mahfouz et al. (2015) discovered modules of such genes with neurobiologically pertinent co-expression dynamics, enriched for functional ontologies related to synaptogenesis and GABAergic neurons. Within the interaction networks identified by Mahfouz et al., a number of hub genes were detected, including *PROCA1*, *TBC1D22B*, *PPP2R2D* and *HACE1*. Other potential gene of interest is *PDGFRB*, which encodes the subunit β of the receptor of the platelet-derived growth factor (PDGF), a potent mitogen involved in the development of the central nervous system. Both *PDGF* and *PDGFRB* have been associated with ASD (Kajizuka et al., 2010). PDGFR-β KO mice show reduced auditory-evoked *γ* oscillation plausibly resulting from reduced number of GABAergic neurons, as observed in the amygdala, the hippocampus, and the medial prefrontal cortex, which in turn give rise to problems with social interaction and spatial memory (Nguyen et al., 2011; Nakamura et al., 2015). Thus, phase-locked *γ* oscillations could be a useful physiological biomarker for ASD (see Nakamura et al., 2015 for discussion).

On a related note, if our model of Dynamic Cognomics is accurate, mutations in genes involved in establishing connections between the cortex and the basal ganglia may also lead to particular aspects of ASD. Among them, we wish to highlight *NLGN1* and *SHANK3*. *Nlgn1* knockout mice exhibit repetitive behaviors and abnormal corticostriatal synapses (Blundell et al., 2010). In turn, *SHANK3* is expressed in the basal ganglia, and when knocked out in mice it leads to abnormal social interactions and also to repetitive grooming behavior (Peça et al., 2011). SHANK3 is a postsynaptic scaffolding protein which appears to be crucial for the maintenance of functional synapses as well as the adequate balance between neuronal excitation and inhibition. Interestingly, among the factors that seemingly modulate this aspect of brain function are the genes controlling circadian rhythms (Bourgeron, 2007). Another gene of interest is *NAV1.1* which encodes a sodium channel. In mice *Nav1.1* down-regulation in the medial septum and diagonal band of Broca dysregulates hippocampal oscillations and results in a spatial memory deficit (Bender et al., 2013).

### ASD-Candidates and Language Evolution

A more systematic account of genes related to language deficits in ASD may emerge from the new directions for exploring the genetic basis of language-readiness in humans proposed in Boeckx and Benítez-Burraco (2014a,b) and Benítez-Burraco and Boeckx (2015a). In these articles, it is noted that the unusually globular braincase of anatomically modern humans (AMH) may have led to changes in the wiring patterns and oscillatory behavior of the hominin brain. Specifically, an increase of cortical matter across anterior and posterior sites is expected to have followed the observed changes in the skull. In turn these changes may have provided the hominin brain with greater working memory resources and ultimately, with enhanced cross-modular connections (being mediated by the more central role played by the thalamus as a strong modulator of fronto-parietal activity and a connector of distant areas). This new neuronal workspace resulting from globularization also involved the cerebellum and particularly, the corpus callosum (Theofanopoulou, 2015), since interhemispheric integration is crucial for language (Poeppel, 2012). The AMH-specific rewiring of the brain had allowed us to transcend (better than other species) the signature limits of core knowledge systems and thus go beyond modular boundaries (Mithen, 1996; Boeckx, 2011). As discussed in Boeckx and Benítez-Burraco (2014a), our language-readiness (that is, our species-specific ability to learn and use languages) boils down to this enhanced cognitive ability. However, for language to exist this ability has to be further embedded inside the cognitive systems responsible for interpretation and externalization (Figure 1). Importantly, as noted in “From Brain Rhythmicity to Language Deficits in ASD” Section, this embedding involves the embedding of high frequency oscillations inside oscillations operating at a lower frequency (see Boeckx and Benítez-Burraco, 2014a for details). This is why we expect that the emergence of our language-also readiness involved new patterns of long-distance connections among distributed neurons and thus new patterns of brain rhythmicity, aspects that are disturbed in ASD.

**Figure 3:**
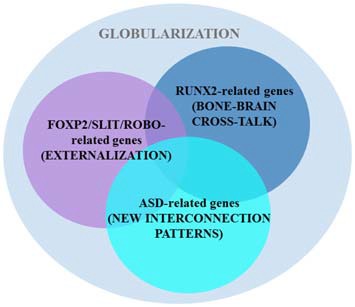
**Several putative gene networks seemingly account for the emergence of language-readiness in our species, including one clustered around AUTS2 and other strong ASD-candidates.** As noted in the main text, two other sets of genes (centered on RUNX2 and FOXP2/ROBO, respectively) also encompass some candidates for ASD (based on Boeckx and Benítez-Burraco, 2014a,b; Benítez-Burraco and Boeckx, 2015a).

Boeckx and Benítez-Burraco put forward a putative gene network (involved in skull morphogenesis and thalamic development, but also in the regulation of GABAergic neurons within the forebrain) that was hypothesized to have been modified after our split from Neanderthals and Denisovans, providing the scaffolding for our species-specific mode of cognition. The network is centered around *RUNX2*, a gene showing strong signals of a selective sweep after our split from Neanderthals (Green et al., 2010; Perdomo-Sabogal et al., 2014). Nonetheless, it also encompasses some *DLX* genes (*DLX1*, *DLX2*, *DLX5*, and *DLX6*) and some *BMP* genes (*BMP2* and *BMP7*; see Boeckx and Benítez-Burraco, 2014a for details). This network is assigned the role of determining parts of language’s syntax-semantics interface. However, it is functionally related to the FOXP2 and ROBO/SLIT interactomes that are claimed to be involved in the externalization of language (see Boeckx and Benítez-Burraco, 2014b for details; Figure 3). Overall, the FOXP2-ROBO/SLIT-RUNX2 connections are interpreted as the result of an evolutionary convergence between the ancient externalization component and the emerging conceptual-intentional component (although see Balari and Lorenzo, 2015 for an argument that these latter networks are more heavily involved in the computational system).

Interestingly, changes in some ASD-candidate genes have been selected in AMHs after our split from extinct hominins, paradigmatically, in *AUTS2*, a gene that according to Green et al. (2010) displays the strongest signal of positive selection in AMHs compared to Neanderthals. Importantly, some of the candidates for ASD belong to the two set of genes highlighted above, including *CTNNB1*, *HRAS*, *DLX1*, *DLX5*, *PTEN*, and *SMURF1* (which are part of the set centered on RUNX2), and *ROBO2*, *FOXP1*, *POU3F2*, and *CNTNAP2* (which belong to the second set of genes; see Benítez-Burraco and Boeckx, 2015b for details), whereas some other candidates (including *AUTS2*, *PAX6* and some of its partners, like *TBR1* and *FEZF2*) provide additional links between the RUNX2 and the ROBO-FOXP2 interactomes (Figure 3; see Benítez-Burraco and Boeckx, 2015a for details). Because the involvement of these three sets of genes in both interfaces of language, this overlapping may account for the observed deficits in ASD regarding language abilities (see Benítez-Burraco and Boeckx, 2015b for discussion). Furthermore, some of the candidates for the ASD dynome interact with some of the genes encompassing these interactomes. For example, according to Kuhlwilm et al. (2013) the expression of both *PDGFRB* and *NLGN1* changes after RUNX2 transfection.

We wish to end by highlighting several similarities between the presumed Neanderthal head/brain/mind and the observed ASD phenotype that may be explained by the evidence presented above (and overall, by all these changes and new connections that contributed to the emergence of language-readiness in our species). As noted, the Neanderthal brain(case) was more elongated; the temporal pole, the orbitofrontal cortex, and the olfactory bulbs were smaller (Bastir et al., 2011); and they didn’t show a uniform parietal surface enlargement (Bruner, 2010). The fact that ASD often appears alongside an abnormal head shape and higher rates of macrocephaly (Lainhart et al., 2006; Cheung et al., 2011), resulting from enhancements in frontal white matter and minicolumn pathology (Casanova et al., 2002; Vargas et al., 2005) speaks to the present globular perspective on linguistic computation, with our species-specific skull morphology influencing our distinctive brain wiring and cognitive abilities, including language. This perspective goes back to Kanner’s (1943) seminal insights concerning individuals with ASD often having “intelligent” and “pensive” physiognomies. This overgrowth results in the forms of frontal over-connectivity discussed above, and stymies the enhancement of anterior to posterior brain region synchronization (Supekar et al., 2009; Coben et al., 2013, 2014). Moreover, as also noted above, both interhemispheric connectivity and language abilities are altered in ASD (Verly et al., 2014). ASD has been characterized in terms of a hyper-modular mind (Kenett et al., 2015) that lacks the cognitive flexibility observed in non-affected people. Interestingly, the Neanderthals mind has been described as a conglomerate of specialized intelligences lacking the cognitive flexibility of AMHs (Mithen, 1996, 2005). All of this does not suggest that ASD is an atavistic trait. However, the study of the etiopathogenesis of ASD (including candidate genes and abnormal brain rhythms) may benefit from ongoing studies of language evolution in the species (and vice versa). Recent research has linked the emergence of complex, highly prevalent conditions like ASD to the uncovering of cryptic genetic variation resulting from our evolutionary history (Gibson, 2009).

Finally, we wish to note that under the globularity hypothesis, visual abilities are claimed to be different in humans and Neanderthals, plausibly because of the selected differences in *PAX6* and related genes (see Benítez-Burraco and Boeckx, 2015a for discussion). It is of interest, then, that atypical visual processing in higher cognition has been extensively documented in ASD, with a general increased reliance on visual imagery perhaps being a compensatory mechanism in lexical processing. For instance, greater occipital activation is seen in ASD compared to TD controls during embedded figure tasks, where subjects must detect geometric figures within a larger visual pattern (Ring et al., 1999). Baron-Cohen et al. (2005) also document enhanced visual-spatial skills relative to verbal skills. However, Bertone et al. (2003) presented evidence that the otherwise high visual integration abilities of subjects with ASD degenerate when second-order visual information is introduced, suggesting that any high performance visual skills they have are limited to basic, non-hierarchical stimuli. Problems with hierarchies also appear to arise in the performance of people with ASD during the perception of biological motion and facial masks (Blake et al., 2003; Deruelle et al., 2004). Relatedly, the resistance of people with ASD to McGurk effects (McGurk and MacDonald, 1976), implicating speech and facial articulatory integration, may possibly be explained not by invoking social comprehension skills, but rather by pointing to their impaired facial-speech hierarchical predicting abilities. Broadly speaking, “autistic perceptual processes are primarily not hierarchical, favoring fragmentary over holistic processing” (Bourguignon et al., 2012: p. 139). While word-level comprehension is either intact or enhanced in ASD (likely a consequence of the increased posterior temporal and occipital activation noted above), sentence-level hierarchical processing is typically impaired, with superior visual processing being insufficient for the processing of hierarchical syntactic principles like c-command (involved in binding relations between pronouns and their antecedents) and A-movement (involved in the formation of passives; Perovic et al., 2007). This suggests a more general deficit in hierarchical processing in ASD, which, under the present cognome-dynome model, is a consequence of oscillatory impairments and the resultant coupling restrictions. ASD also often leads to reduced verbal imagination and inner speech (Whitehouse et al., 2006), along with reduced symbolic play (Honey et al., 2006). Fries (2015: p. 232) documents that visual scenes induce multiple *γ* rhythms with varying frequencies, yielding a wide “gamma landscape” which, we believe, the enhanced visual cognition of autistic individuals can easily find its place. In contrast, the landscapes of linguistic computation rely on the coupling of a range of frequency fields, leaving certain properties of linguistic cognition susceptible to disruption.

## Conclusion

**Figure 4:**
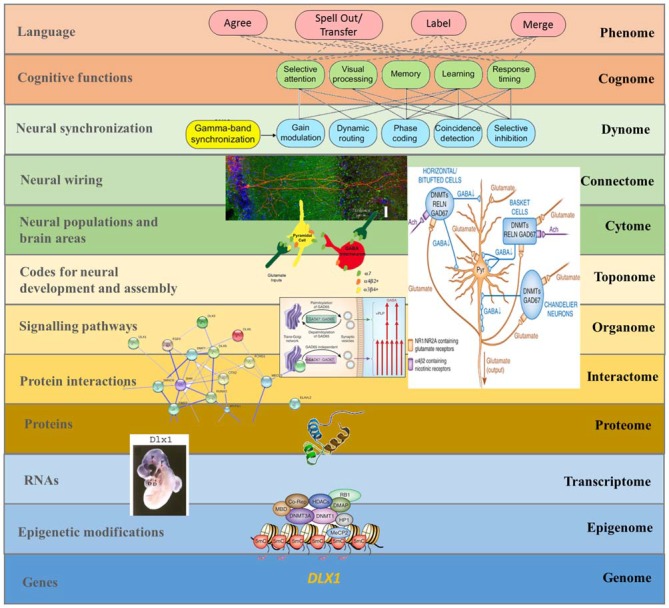
**A multilevel approach to language deficits in ASD from an oscillatory perspective.** Understanding language problems in ASD demands a systems biology approach that seeks to unravel the nature and links between all biological factors involved. The figure shows one possible line of research focused on brain oscillations, although many others will need to be explored in the future to gain a comprehensive view of this complex issue. As noted in the main text, among the candidates for ASD we find several genes that have changed during recent human evolution and that are believed to be important for the emergence of language. One of them is DLX1, known to control aspects of skull and brain development. As discussed in Boeckx and Benítez-Burraco (2014a), DLX1 is expressed in neocortical GABAergic neurons and it regulates thalamic differentiation and interconnection with the cortex. As the String 9.1 network shows, DLX1 interacts with other core candidates for globularization, like RUNX2 and DLX2. RUNX2, DLX1, and DLX2 are key components of the GAD67 regulatory network, which is important for the normal development of GABAergic neurons within the hippocampus. As noted in the text, disturbances in GABAergic mediator system may contribute to the altered γ activity detected in the hippocampus of ASD children, which may impact on syntactic operations in ASD language. The expression pattern in the forebrain of transcription factors like DLX1 (here exemplified with an *in situ* hybridization of the Dlx1 in E10.5 mouse embryo) has changed over the course of our history. This may explain some of the changes that reshaped our species-specific program for the generation of neocortical local circuit neurons and, ultimately, the changes in GABAergic input to several brain areas (including the hippocampus). In turn, this may have contributed to the retuning of brain oscillations that brought about modern cognitive functions like language (at the top of the figure), although the exact role played by these basic cognitive operations in language processing is still unknown. This, then, is a composite figure elaborated by the authors. The dynomic-cognomic aspects of γ-oscillations (on the top of the figure) are from Bosman et al. (2014). The micrography of the single hippocampal CA1 pyramidal neuron is from http://basulab.us/research/goals. The schematic view of the hippocampal GABAergic neurons (below) is from Feduccia et al. (2012). The scheme of the two distinct mechanisms targeting GAD67 to vesicular pathways and presynaptic clusters is from Kanaani et al. (2010). The String 9.1 network is from Boeckx and Benítez-Burraco (2014a). The schematic view of the structure of the DLX1 protein has been taken from www.uscnk.com. The* in situ* hybridization of Dlx1 in E10.5 mouse embryo is from Panganiban and Rubenstein (2002). The schematic representation of a transcriptionally inactive promoter is from Grayson and Guidotti (2013). Finally, the large scheme on the right of the picture shows the effect of DNA methyltransferase overexpression on GABAergic neurons and is also from Grayson and Guidotti (2013) (of interest is that GABAergic promoter downregulation is observed in some cognitive disorders like schizophrenia, resulting in increased levels of some DNA methyltransferases like DNMT1 and 3A, and reduced GAD67, RELN and a variety of interneuron markers).

Overall, these considerations may provide a suitable response to Dehaene et al. (2015: p. 2) observation that linguistic computation requires “a specific recursive neural code, as yet unidentified by electrophysiology, possibly unique to humans, and which may explain the singularity of human language and cognition”. Our “rhythmic” project will be long-lasting, encompassing a top-down approach to language processing in the brain, from linguistic features to brain rhythms to genetics (Figure 4), but we wish to highlight that it encompasses a number of neurodegenerative and neurodevelopmental disorders that should help yield insights into the structure and function of language and mind. In fact, we expect disorders to be particular areas within the whole morpho-space or adaptive landscape of language development in the species, defined by the site of basic brain rhythms (see Benítez-Burraco, 2016 for discussion). As we have argued, ASD is of particular interest in virtue of it representing a mode of cognition and perception distinct from, but plainly related to, normally functioning linguistic cognition.

We further expect that the present perspective of Dynamic Cognomics has the potential to provide robust endophenotypes of ASD. Importantly for ongoing research into the biological underpinnings of ASD, the process of generating and testing dynomic predictions will permit the falsification of a large number of possible dynome-cognome linking hypotheses, allowing the refinement of the present model of ASD, while simultaneously granting more comprehensive and earlier diagnoses of language deficits in this condition. We expect to extend this perspective to other conditions such as schizophrenia, which has been claimed to be at the opposite pole to ASD within a continuum of modes of cognition also encompassing TD cognition (see Crespi and Badcock, 2008 for discussion). Interestingly, the abnormal *γ* documented in schizophrenics by Xu et al. (2013) is likely a cause of the problems with producing pronounceable nonwords and confusion of antonyms seen in this disorder (Stephane et al., 2007), in contrast to this rhythm’s implication in over-connectivity in ASD. Inner speech is also potentiated in schizophrenia through auditory verbal hallucinations, unlike in ASD (Moseley et al., 2013). Moreover, a thalamocortical “dysrhythmia” has been documented in subjects with schizophrenia, obsessive-compulsive disorder and depression by Schulman et al. (2011). Evidence from EEG, MEG and anatomical studies suggests that oscillatory synchronization abnormalities may play a core role in the pathophysiology of schizophrenia (Uhlhaas and Singer, 2010). In particular, synchronization between *γ* and *β* appears to be abnormal in several studies examining visuo-perceptual organization and auditory processing (Spencer et al., 2003; Symond et al., 2005; Uhlhaas et al., 2006), while *γ* activity displays severe widespread deficiency during perceptual organization tasks (Tillmann et al., 2008). Overall, these findings suggest a distinct oscillopathic profile from that of ASD.

To conclude, we wish to note that these considerations also speak against the newly emerging view in the literature (Berwick and Chomsky, 2016) that language evolution will either remain a mystery or should be explored at the neurological level purely through functional localization studies. As discussed, abnormal cognitive/linguistic development in our species should help unravel the evolutionary path followed by our faculty of language, as the high number of candidates for ASD selected in AMH nicely illustrates. In this respect, schizophrenia is again a natural target (see Berlim et al., 2003 or Crow, 2008 for discussion). It remains to be seen how far the present dynomic model of linguistic computation can be used to enhance understanding of other language-related oscillopathies.

## Author Contributions

Both authors contributed to all sections of the article.

## Conflict of Interest Statement

The authors declare that the research was conducted in the absence of any commercial or financial relationships that could be construed as a potential conflict of interest.
